# Predictive Value of CTA Spot Sign on Hematoma Expansion in Intracerebral Hemorrhage Patients

**DOI:** 10.1155/2017/4137210

**Published:** 2017-08-09

**Authors:** Wen-Jie Peng, Cesar Reis, Haley Reis, John Zhang, Jun Yang

**Affiliations:** ^1^Department of Neurology, The First Affiliated Hospital of Chongqing Medical University, 1 Youyi Road, Chongqing 400016, China; ^2^Department of Physiology and Pharmacology, Loma Linda University School of Medicine, 11041 Campus Street, Risley Hall, Room 219, Loma Linda, CA 92354, USA; ^3^Department of Neurosurgery, Loma Linda University School of Medicine, Loma Linda, CA 92354, USA

## Abstract

Hematoma expansion (HE) occurs in approximately one-third of patients with intracerebral hemorrhage and leads to high rates of mortality and morbidity. Currently, contrast extravasation within hematoma, termed the spot sign on computed tomography angiography (CTA), has been identified as a strong independent predictor of early hematoma expansion. Past studies indicate that the spot sign is a dynamic entity and is indicative of active hemorrhage. Furthermore, to enhance the spot sign's accuracy of predicting HE, spot parameters observed on CTA or dynamic CTA were used for its quantification. In addition, spot signs detected on multiphase CTA and dynamic CTA are shown to have higher sensitivity and specificity when compared with simple standardized spot sign detection in recent studies. Based on the spot sign, novel methods such as leakage sign and rate of contrast extravasation were explored to redefine HE prediction in combination with clinical characteristics and spot sign on CTA to assist clinical judgment. The spot sign is an accepted independent predictor of active hemorrhage and is used in both secondary intracerebral hemorrhage and the process of surgical assessment for hemorrhagic risk in patients with ischemic stroke. Spot sign predicts patients at high risk for hematoma expansion.

## 1. Introduction

**Figure 1:**
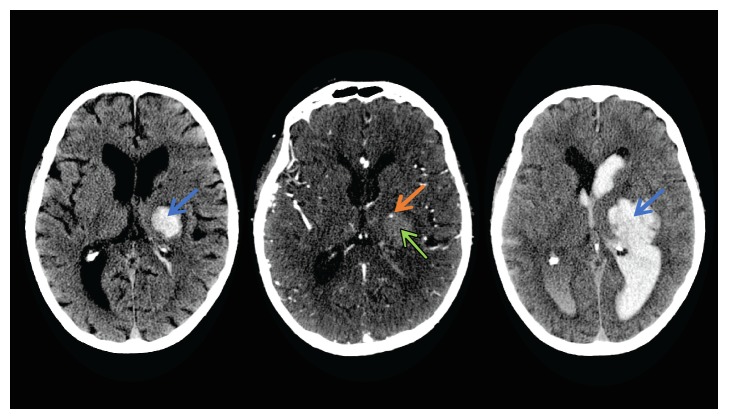
These images depict a left hemisphere ICH. A single sign with a spot-like appearance on CTA (orange arrow). The hyperdense area (blue arrow) in the first picture signifies an early hematoma in a baseline noncontrast CT. The second image shows that a spot sign (orange arrow) within the hematoma (green arrow) is discontinuous to any outside vessels and has a higher CT HU than the background hematoma in real CTA. The third image shows an expanding hematoma (blue arrow) in a 24 h follow-up noncontrast CT. Image provided by Dr. Andrew M. Demchuk, M.D., FRCPC.

Spontaneous intracerebral hemorrhage (ICH) accounts anywhere from 10% to 50% of overall acute strokes depending on the population, race, and region being studied. It is the most severe subtype of stroke with a high mortality rate of ranging from 35% at 7 days to 59% at 1 year [1–7]. The primary injury of ICH is mainly attributed to the mass effect of hematoma, with the secondary injury mechanism being somewhat unclear [8]. With early neurological deterioration, the devastating complications of ICH are associated with large initial ICH volumes and early hematoma expansion (HE) after the first few hours of ICH onset [2, 6, 9, 10]. Approximately 33% of patients with ICH are destined to suffer HE, and this has been recognized as the major cause of mortality and morbidity. Therefore, it is of key importance to predict HE in order for clinicians to perform timely therapeutic strategies to help reduce the morbidity and mortality associated with hematoma expansion. Spot sign, first proposed in 2007 and described as a 1-2 mm sized foci of contrast enhancement or the presence of high-density material on CTA within an acute primary hematoma no matter its shape [2], has been demonstrated to predict HE following acute ICH. The definition of spot sign was found inconsistent in different studies [2], but almost all studies agree that spot sign indicates accumulation of leaked contrast medium. Although the biological underpinnings of HE remain unclear, a culmination of evidence supports a model of ongoing secondary bleeding from ruptured vessels adjacent to the initial bleeding site [4, 11], directly reflecting the process of spot sign (Figure 1). However, not all positive spot signs represent ongoing bleeding [9]. Several vascular and nonvascular mimickers of CTA spot sign have been identified, as well as ways to avoid misinterpretation [9, 12].

Describing the dynamic evolution of spot sign aids in understanding the pathology of spot sign [4] and distinguishing truly at-risk patients. A systematic review and meta-analysis evaluating the accuracy of spot sign show that the studies of first-pass (static) CTA identified spot sign with a sensitivity of 53% (95% CI, 49%–57%) and a specificity of 88% (95% CI, 86%–89%). The pooled positive likelihood ratio (PLR) was 4.70 (95% CI, 3.28–6.74) and the negative likelihood ratio (NLR) was 0.44 (95% CI, 0.34–0.58) [13]. A similar systematic review regarding the accuracy of spot sign in recent years searched 259 related studies of CTA spot sign from 1980 to May 2012. Only 6 studies satisfied inclusion criteria that all spontaneous ICH patients were evaluated by CTA, received a follow-up CT, and reported clinical outcome accuracy measures of spot sign in predicting HE. The definition of spot sign or bad clinical outcome was not clear in some studies and inconsistent in all studies. Finally, the high variability found in this review on CTA spot sign studies made it difficult to conclude that spot sign was a promising marker in predicting HE [2]. The results are a reminder that a large number of spot sign negative patients may miss early diagnosis and effective interventions and spot sign positive patients may not undergo HE. Improving the diagnostic accuracy of the spot sign for the prediction of HE has been the focus of recent studies. In addition to CTA, imaging tools including magnetic resonance imaging (MRI), computed tomography perfusion (CTP), and contrast-enhanced CT are being explored for their use in detecting spot-like signs. These trials will be helpful in evaluating clinical prognosis using spot sign as diagnostic criteria, as well as providing evidence and future directions for ICH studies.

## 2. Hematoma Expansion

Initial hematoma expansion following spontaneous acute ICH is an important marker of poor prognosis, increased mortality, and longer hospital stay. After the first year, more than three-quarters of the patients with primary ICH are severely disabled or deceased. Several risk factors are related to HE, including baseline ICH volume, hematoma location, early presentation after symptom onset, anticoagulation, and the spot sign on CTA [4]. Initial hematoma volume remains the strongest predictor of 30-day mortality, and functional outcomes are connected with both short- and long-term outcomes [4, 14]. However, even when the initial volume is small, the patient is still at risk for hematoma expansion and subsequent poor outcomes. The process of hematoma growth was described in an alternative “avalanche” model for HE proposed by Fisher in the early 1970s. The model explains the process of hematoma enlargement as secondary mechanical shearing of neighboring vessels caused by expansion of the initial hemorrhage, which is directly supported by the interpretation of CTA spot sign representing a site of active bleeding (visualized as contrast extravasation following venous contrast injection) [4, 11]. However, early evacuation of hematoma and conservative treatments including controlling blood pressure or reversal of coagulopathy provided minimal clinical benefits, as inflammation becomes the main contributor of secondary brain injury in the early stages (Figure 4) [15, 16]. The metabolic products or components of hematoma, such as hemoglobin, heme, and iron, also lead to the formation of secondary hematoma and brain injury [4, 17, 18]. HE was defined differently across studies using relative (e.g., >30% or 33%) or absolute change (e.g., mostly >6 or >12.5 ml) in hematoma volume from baseline CT to follow-up CT [13, 19] or a combination of both [4]. The frequency of HE in the studies was influenced by the variations in definition of HE, the timing of symptom onset to initial CT, different volumetric assessments, and clinical outcomes. HE is considered a hyperacute phenomenon and is associated with early neurological deterioration (defined as having a greater score of 4 points or more on the NIHSS scoring system at 24 hours compared to baseline) [10, 20, 21]. HE generally occurs within 3 h of symptom onset, while a substantial subset of all expanders (up to 48%) present at least six hours after ICH ictus [4, 14, 22]. However, with each 10% increase in size of initial ICH, there is a 5% increase in mortality and additional 16% chance of poor outcome [23]. Stability of initial hematoma in the first week entails a good prognosis. HE is the only biological marker of outcomes, making the prediction of potential HE crucial for garnering positive clinical outcomes.

## 3. Spot Sing on CTA

**Figure 2:**
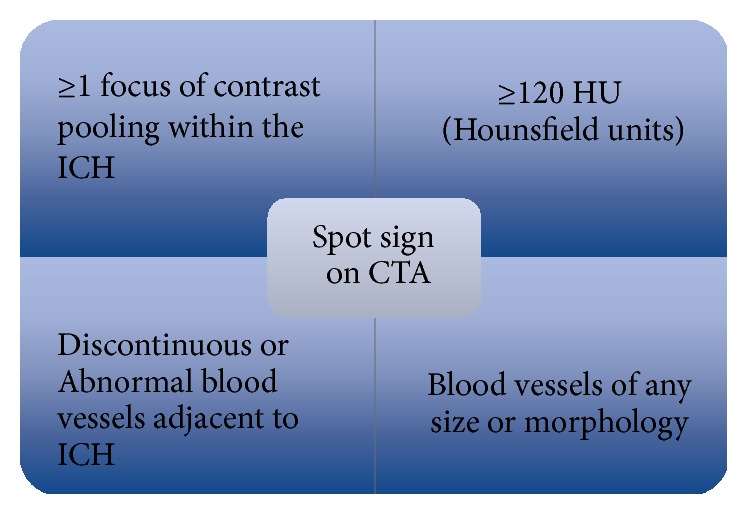
This schematic highlights several important parameters in detecting spot sign.

**Figure 3:**
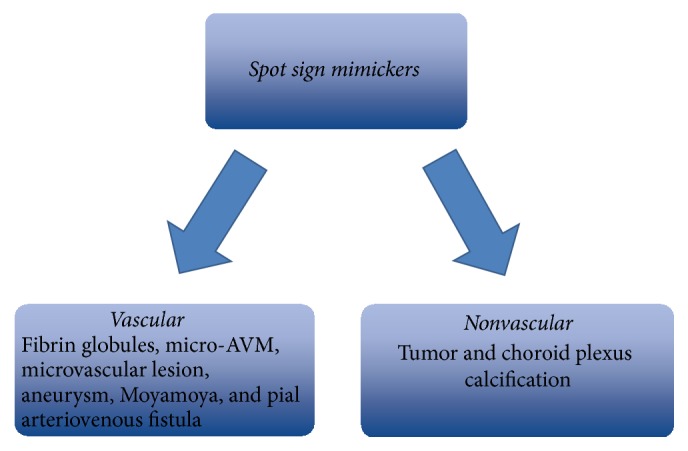
This graphic represents 8 other causes for spot sign on CTA, vascular mimickers (fibrin globules, micro-arteriovenous malformation (micro-AVM), aneurysm, microvascular aneurysm, Moyamoya, and pial arteriovenous fistula) and nonvascular mimickers (tumor and choroid plexus calcification). This suggests that not all positive spot signs represent ongoing bleeding and clinical judgment and other diagnostic tools should be utilized.

In recent years, the use of CTA in emergency situations has increased, as it is noninvasive, low-risk, and a fast diagnostic tool. Multiple studies found that patients with spot sign on CTA are more likely to experience HE after primary acute ICH than those without [20, 21, 24–26]. The spot sign was defined using the following criteria: (1) ≥1 focus of contrast pooling within the ICH; (2) an attenuation ≥120 Hounsfield units (HU) (approximately double the background hematoma density); (3) being discontinuous from normal or abnormal blood vessels adjacent to the ICH; and (4) blood vessels of any size and morphology, which can be easily identified by nonradiologists (Figure 2) [27]. Those vessels entering the hematoma from the periphery and connecting with the region of contrast extravasation should not be confused with the hematoma [28]. Apart from the site of active extravasation, a locus of arrested hemorrhage-forming fibrin globules or associated epiphenomena such as hypertensive microaneurysms from a microvascular lesion also present as a “spot sign” on CTA (Figure 3). As a leading pioneer in describing ischemic stroke and publishing extensive research in the field of neurological diseases, Dr. Fisher suggests that fibrin globules represent concentric fibrin rings surrounding masses of red blood cells from a small ruptured artery [29]. The manifestation of vascular mimickers (fibrin globules, micro-AVM, aneurysm, Moyamoya, and pial arteriovenous fistula) and nonvascular mimickers (tumor and choroid plexus calcification) is consistent with spot sign, including a diameter ranging from 0.3 to 1 mm and high-density appearance on CTA. Sometimes it is difficult to recognize all the mimics from a static spot sign. However, the mimickers can grow or diffuse into the hematoma according to the dynamic CTA and therefore could not be mistaken as active extravasation [9]. As we know, digital subtraction angiography (DSA) is the gold standard for confirmation of vascular lesions. A recent study comparing CTA with digital subtraction angiography in nonhypertensive patients younger than 45 years old determined high negative and positive predictive values for CTA (97.3% and 100%, resp.) in establishing vascular etiology of ICH and distinguishing the secondary ICH at the same time [7]. DSA can be used to aid in identification of the vascular source and avoid misdiagnosing a spot sign mimicker for an ICH spot sign. A study by Gazzola and colleagues identified CTA spot sign mimickers, from both vascular and nonvascular origins, in patients with secondary intracerebral hemorrhage. Nonvascular mimics identified in their study included underlying neoplasms causing calcification in associated peripheral hyperdense regions, as well as calcific deposition in the choroid plexus. Careful examination of the choroid plexus on subsequent noncontrast CT images helped avoid misinterpreting the hyperdense region. They also suggested physiological and postinfective or inflammatory calcification as potential mimickers of spot sign. Vascular mimics included partially thrombosed posterior communicating aneurysms, microaneurysms, and Moyamoya. In these cases, it was important to carefully identify the location of hyperdense areas, particularly with regard to whether they were within or external to the hematoma and brain parenchyma [12].

A prospective observational study in 2012 called PREDICT demonstrated that CTA spot sign can identify a subpopulation of patients with ICH who are at high risk of substantial hemorrhage. In those patients with HE, sensitivity for spot sign was 63% and specificity was 90%, with a positive predictive value (PPV) of 73%, and a negative predictive value (NPV) of 84% [1]. Mortality at 3 months was 43.4% in CTA spot sign positive group versus 19.6% in CTA spot sign negative group [25]. However, the latest study in 2016 identified the spot sign's NPV increased to 98% [30]. Spot sign on CTA is a strong predictor of underlying vascular lesions and HE. In order to systematically characterize the spot sign to better identify features most predictive of HE, a spot sign scoring system was constructed. The amount of contrast pooling, dimension, and Hounsfield units of spot sign were confirmed as significant features [21]. In multivariate analysis, the spot sign score (SSS) was the strongest predictor of significant HE and poor outcomes among survivors at three-month follow-up [31], independent of time from ictus to CT angiogram evaluation [21]. Polycentric external validation of the SSS and the use of spot sign demonstrated that the spot sign number alone provided similar prediction but improved risk stratification of HE compared with the SSS [30, 32]. Additionally, a study reported the positive predictive value for significant expansion increased with number of spot signs. When four spot signs were detected, the patient suffered HE and poor clinical outcomes. The study's outcomes confirmed a strong association between spot sign and HE [9]. It is evident that early spot sign detection can help prevent worsening outcomes associated with hematoma expansion. Correctly identifying spot sign is an important issue. Although rate of contrast injection, contrast dose, and volume can be standardized, varying blood pressure, circulation time, and perihematoma intracranial pressure changes influence the time to spot sign visualization and its yield [3]. It is unlikely that a single optimal CTA time for spot sign delineation exists [3]. A case report in 2009 showed that spot sign only appeared in later images through time-resolved dynamic CTA (dCTA). The dynamic sequence of spot sign appeared at 34 s, 39 s, 44 s, 49 s, and 60 s [23]. In 2011, another study clearly demonstrated an increasing area of contrast collection seen along the anterior lateral periphery of the hemorrhage consistent with a spot sign. The spot first appeared at 15 s following start of contrast injection and steadily increased in size and density. The maximum density was noted around 25 s. Following this, the average density decreased with the spot appearing more heterogeneous and the margins became ill-defined, consistent with dispersion of contrast in the hematoma [33].

Contrary to standard “static” images that are acquired at a particular point in time, dCTA demonstrates temporal washin and washout of intravenous contrast material and captures evolution of the spot sign in real time [23, 33]. It is consistent with other studies affirming that the evolution of spot sign by dCTA represents a site of active bleeding [9]. Spot sign identified in early stages of HE indicates a site of temporal active extravasation, with later spot sign representing resolved hemorrhage after physiological hemostasis or tamponade from rising intracranial pressures [9]. Additionally, dCTA was used to describe spot sign characteristics and measurement parameters over 60 seconds of image acquisition [34]. Chakraborty and colleagues described the evolution of spot sign through relative parameters including the earliest appearance, spot duration, maximum Hounsfield unit (HU), time to maximum HU, time to spot sign diagnostic criteria (based on >100 or >120 HU), and the linear rate of spot sign formation (the change in volume between its first visualization and the phase when it has reached the maximum density divided by time). The article acknowledges how spot sign is a temporally evolving phenomenon rather than a simple dot in hematoma (Figure 1) [34]. Founded on this evolving theory of spot sign, further studies determined that the detection frequency of spot sign might be increased in the venous phase of CTA compared to the arterial phase [9], improving the sensitivity of spot sign from 55% to 64% on a 90-second CTA [15]. The interval time between the venous and arterial phase made spot sign sensitivity different [35]. However, adequate timing of additional or delayed CTA and which option is the most effective in improving the accuracy of spot sign in predicting HE are controversial. In addition, increasing the detection rate of spot sign does not mean increasing the positive predictive value of spot sign in HE. To some extent, the frequency of detecting HE influences the accuracy of spot sign, as spot sign is a prognostic index and HE is the end result. HE presenting within 6 hours of symptom onset occurs in up to 48% patients, with the remaining patients presenting with HE either late or with an unknown symptom onset time [22]. The CTA spot sign accurately identifies patients destined to expand regardless of time from symptom onset, and predicting HE thus appears to be an important goal even in patients with late presentation [4, 22].

## 4. Emphasizing Factors

Specific factors correlated with spot sign were tested to improve the predictive value of CTA spot sign in HE. After dividing ICH patients into expansion and nonexpansion groups, Kim and colleagues concluded that the conditions of shorter time from symptom onset to initial CTA and the higher HU of spot sign are the emphasizing factors of spot sign for predicting HE [36]. Although it is too early in some cases to allow contrast accumulation within hematoma, especially in those patients with lower cardiac output or higher peripheral vascular resistance, spot sign observed in earlier phases may be associated with greater absolute enlargement [9, 36]. A more recent study shows that less time between CT perfusion and first detection of spot sign greatly improves the specificity of HE [37, 38]. The authors redefined the spot sign based on timing of contrast leakage on CTP and found that a spot sign showing before 23.13 seconds improves the specificity of the spot sign for predicting HE as well as the 3-month mortality after secondary ICH [39]. The timing of occurrence of CTP spot sign may reflect the rate and volume of ongoing bleeding in hematoma. In short, the use of early-occurring spot sign might improve the positive predictive value of spot sign. The higher HU of spot sign reflects more accumulation of escaped contrast agent and is associated with absolute HE. When the cut-off value of HU was set at 171 in the ROC curve measurement, the sensitivity and specificity for HE were 65.4% and 85.7%, respectively. The sensitivity of data was 100% when the absolute HU was over 191.0 [36]. There are technical factors that contribute to the identification and quality of spot sign. Reduction of tube current is an effective strategy to minimize radiation load; however, a new study recruited 709 patients and classified them into two groups (low current and high current) and identified that CTA obtained at high levels of tube current could ensure better image quality and diagnostic detection of spot sign [40]. In addition, genetic factors, particularly presence of apolipoprotein E (APOE) alleles affect the appearance of spot sign. A multicenter genetic association study led by the International Stroke Genetics Consortium demonstrated associations between the APOE*ε*2 allele and larger baseline ICH volumes and HE. The role of the APOE alleles is predominantly associated with vasculopathic changes ultimately leading to rupture of the diseased vessels, whereas *ε*4 increases the severity of amyloid deposition within the vessel wall [41]. Those who possess the APOE*ε*2 allele are more likely to have spot sign.

## 5. Modified Spot Sign

**Figure 4:**
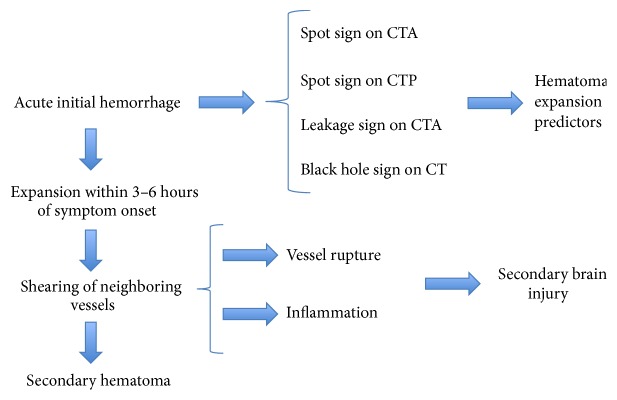
This figure demonstrates the process of hematoma expansion and its secondary effects. Spot sign on CTA (computed tomography angiography) and CTP (computed tomography perfusion) and leakage sign on CTA are strong predictors of hematoma expansion. Utilizing spot sign in acute initial hemorrhage can help reduce secondary brain injury and inflammation and thus reduce morbidity and mortality associated with hematoma expansion.

Spot sign has been identified to be a dynamic entity, thereby generating novel methods to aid in predicting HE. A modified spot sign, called the leakage sign, was evaluated to be a better predictor of HE after ICH (Figure 4). After the first CTA, researchers performed another scan 5 minutes later (delayed images) and analyzed these two images. To be specific, they detected the same ROI (region of interest) with a 1 cm diameter in both images, which varied at the greatest extent, and a 10% increase of HU in the ROI within the two-phase images, findings consistent with a positive leakage sign or HE. The leakage sign had a high sensitivity (93.3%) and specificity (83.9%) for HE and a higher sensitivity (93.8%) and specificity (91.4%) if united with a spot sign. According to this study, it may be beneficial to get another image after 5 minutes for comparison purposes in patients without respiratory or cardiac problems [42].

Similar to leakage sign, rate of contrast extravasation or spot sign growth has a high predictive value for HE. A 90-second delayed CTA following the first-pass CTA and spot sign volume changes were measured using semiautomated software. Increased median spot sign volume was 0.36 mL between the two-phase CTA. Median extravasation rate was higher among expanders compared with nonexpanders. In multivariable analysis, the extravasation rate was independently associated with in-hospital mortality (odds ratio, 1.09 [95% confidence interval, 1.04–1.18], *P* = 0.004), 90-day mortality (odds ratio, 1.15 [95% confidence interval, 1.08–1.27]; *P* = 0.0004), and HE (odds ratio, 1.03 [95% confidence interval, 1.01–1.08]; *P* = 0.047) [43]. An earlier article published in 2011 mentioned that early rate of contrast could better predict HE. Compared with CTA, CTP tracks a contrast bolus through the intracranial circulation typically for 60–120 seconds with modern biphasic techniques and seems to be a more reliable tool to predict HE. In this study, CTP spot sign presence was an independent predictor of HE and poor outcomes (*P* = 0.040) and demonstrated greater sensitivity (78%) than spot sign detected on CTA (44%, *P* = 0.034) and postcontrast CT (50%, *P* = 0.025). CTP satisfies the need for early and late acquisitions and shows contrast extravasation not present on CTA, reflecting the transient dynamics of spot sign opacification. Perhaps the use of CTP requires further studies and analysis using a larger sample size in order to satisfy its use in determining HE [3, 43]. In emergency situations, CTA and MRI are currently the modalities of choice for detecting HE.

## 6. Discussion and Future Directions

HE after ICH frequently happens within the first six hours after initial ictus, even with medical attention. Prevention and premanagement of HE is more important than treatment. Clinical features including headache, vomiting, and coma are nonspecific symptoms. Therefore, imaging tools play a key role in diagnosing, determining the underlying etiology, and predicting HE in order for clinicians to provide appropriate supportive therapy and targeted novel medical and surgical interventions [6, 7, 15]. In our review, we searched articles regarding predicting HE using PubMed Data and summarized how spot sign-related studies have affirmed that CTA spot sign is an independent and reliable predictor of HE in patients with acute ICH since 2007. The American Heart Association (AHA) guidelines for ICH management have been modified to suggest CTA, CT venography, contrast-enhanced CT, contrast-enhanced MRI (CEMRI), magnetic resonance angiography, and magnetic resonance venography for the assessment of underlying vascular lesions [28]. CTA is easy to operate, widely accessible, and noninvasive. Using CTA requires immediate admission resulting in some limited conditions. Earlier detection of spot sign related to absolute hematoma is also a big challenge for radiologists. An emerging imaging marker on CT, the black hole sign, was identified as a predictor of early HE in 2016. The black hole sign is defined as a relatively hypoattenuated area encapsulated by an adjacent hyperattenuated area with a 28 HU difference between the 2 density regions [28]. This sign originated from the understanding that hematoma heterogeneity is closely related to early HE and a subsequent original investigation identified hypodensities within an acute ICH detected on CT associated with HE. Previous studies found heterogeneity or low attenuation within a hematoma on noncontrast computerized tomography (NCCT), called the “swirl sign,” to be predictive of HE in addition to CTA spot sign [40, 44]. CTA spot sign has a higher HU than the background hematoma density and also refers to hematoma heterogeneity, but there is increasing need to identify the ideal specific HU threshold in swirl sign, spot sign, and black hole sign. A recent study reported that spot sign on MRI could predict HE and has a long-time window of 24 hours. Spot sign with HE was observed in 23 out of 50 (46%) patients with a Kappa = 0.92 (*P* < 0.001). More importantly, T1 weighted images have the same intensity as cerebral parenchyma in hyperacute stages of hematoma; therefore, it has a lower detection rate compared with CTA [45]. Because this study was observational and lacked long-term follow-up, more studies using MRI for predicting HE are needed. Regrettably, first-pass CTA pooled a low sensitivity; however, the time-resolved dynamic spot signs were advocated due to the increased detection rate and sensitivity. With the poor prognosis due to undetected spot sign, it is of great value to acquire an image several minutes later to enhance the yield of spot sign. Furthermore, in a special study of simultaneously detecting risks and benefits of CTA, adverse events related to CTA, including transient renal dysfunction and allergy to contrast medium, completely resolved within 1 week without any permanent deficits [46]. Given the potential benefits and risks, we believe that CTA performed at admission on all patients with ICH, especially in emergency situations, is beneficial in predicting the hematoma. The majority of studies focused on the primary ICH, but secondary ICH, including arteriovenous fistulas, Moyamoya, and elevated admission blood glucose, could be partly predicted with spot sign [47]. Spot sign represents the process of hemorrhage. Apart from primary and secondary ICH, CTA spot signs use in future surgical trials could forecast intraoperative or postoperative rebleeding [46], larger residual ICH volumes in patients undergoing hematoma evacuation, or hemorrhagic patients at risk for spontaneous ICH [5] or could be to assess the hemorrhagic risk after anticoagulants and therapy for ischemic infarction [18].

Though the pathology of spot sign still remains unclear, there is no doubt that spot sign is related to recurrent or active bleeding. These findings allow the use of spot sign to be translated to different areas of medicine, including surgical indications. Despite spot sign success in predicting HE, spot sign has not been viewed as a gold standard to predict HE in ICH patients. To improve the clinical predictive value of spot sign, linking spot sign with other clinical characteristics is a helpful method in improving detection. Anticoagulation therapy might simply lead to a greater phenotypic expression of the spot sign, such as multiple spot signs, but result in greater HE through prevention of clotting. Patients on warfarin were more likely to have a spot sign regardless of ICH location [19]. An animal model of warfarin-associated ICH showed that mice anticoagulated with warfarin had a higher mortality rate after 24 h (14/44) than those without. Clinical factors such as cardiac output, blood pressure, and tumors could influence the recognition of a spot sign. For example, a prediction score composed of warfarin use, shorter time to CT, CTA spot sign, and baseline ICH volume based on the multivariable logistic regression model was developed and the incidence of HE increased steadily with higher scores, reaching 80.0% for patients with the highest score of 9. The prediction score also performed well when in-hospital and 3-month mortality were assessed. To maximize clinical usefulness of the score it was classified into three categories: low (score of 0 and incidence rate of 5.7%), medium (score of 1–3 and incidence rate of 12.4%), and high (score of 4–9 and incidence rate of 36.4%) [48]. A prospective study showed that the NIHSS score or the mRS scores at 90 days are higher in the spot-sign positive groups versus the spot-sign negative groups [1]. Similar multicenter and large-sample clinical trials regarding risk stratification based on spot sign are promising areas for future clinical research [20].

After the initial injury of ICH, ascribed as tissue disruption and mass effects of the hematoma, a list of secondary toxic events occur including physiological responses, release of blood degradation products, and brain iron overload, leading to late severe neurological deficits including edema, breakage of blood brain barrier, and free radical injury [15, 49–52]. From a molecular perspective, oxidative stress, excitotoxicity, and inflammation are known pathophysiological mechanisms of ICH [53]. Differential alternative splicing in RNA from whole blood suggests immune responses differ between ICH and ischemic hemorrhage and remind us to explore the pathology of ICH on a genetic level [54]. Multiple mechanisms mentioned above contribute to neural deficits after ICH. Typical approaches in the use of hemostatic agents, acute blood pressure reduction, and surgical intervention are helpful but have no reduction in mortality [15]. Multimodality managements including CTA plus prothrombin complex concentrate (PCC) for predicting and preventing HE were created and identified to result in overall decrease in the mean hematoma size at 24 h, whereas the control group showed an overall increase [17, 55]. Attenuating the inflammatory response in the early stages to reduce secondary brain injury and keeping a modest inflammation background in the later stages to promote neurogenesis and recovery is another area for exploration. Therapeutic approaches to treat ICH should focus on the perihematoma region surrounding the lesion and how to prevent the damage from spreading [56, 57]. In addition, published studies found that cannabinoid receptor 2 (CB2R) reduced thrombin-induced brain edema and alleviated BBB damage [58]. HE after ICH brings heavy financial burdens and significant medical interventions, and rehabilitation measures are imperative [40]. Aerobic exercise is a catalyst for recovery of neuron damage after ICH [30]. Hyperbaric oxygen therapy (HBOT) combined with other treatments enhance the therapeutic effect significantly regardless of whether the brain injury is acute or chronic [59]. HBOT makes use of increased total atmospheric pressure and partial pressure of oxygen over ambient partial pressures to intervene with genes in cells, including upregulation of trophic and anti-inflammatory genes and downregulation of proinflammatory and apoptotic genes [60]. Evidence suggests HBOT could improve the effectiveness of impaired blood brain barrier and cytotoxic edema following traumatic brain injury and promote the recovery of neurons [61], which is especially fit for hemorrhage recovery. However, HBOT is simply a supplementary means and not a direct treatment of HE. Prediction and prevention of HE are key points in our research.

In conclusion, HE after ICH usually occurs within the first 6 hours of symptom onset. Fortunately, the spot sign on CTA could significantly predict the active hemorrhage and is an independent and reliable predictor of HE in patients with acute ICH. It is related to early neurological deterioration, mortality, and morbidity. The sensitivity of spot sign was 63%, and specificity was 90% on CTA. The dynamic and modified spot signs have higher sensitivity and specificity. Correctly distinguishing the spot sign from mimickers is challenging for clinicians. When a spot sign appears, immediate treatment and close observation are essential.
